# Management of Lupus Nephritis

**DOI:** 10.3390/jcm10040670

**Published:** 2021-02-09

**Authors:** Farah Tamirou, Frédéric A. Houssiau

**Affiliations:** Department of Rheumatology, Cliniques Universitaires Saint-Luc and Institut de Recherche Expérimentale et Clinique, UCLouvain, 1200 Bruxelles, Belgium; frederic.houssiau@uclouvain.be

**Keywords:** lupus nephritis, treat-to-target approach, repeat kidney biopsy, combination therapy

## Abstract

Lupus nephritis (LN) is a frequent and severe manifestation of systemic lupus erythematosus. The main goal of the management of LN is to avoid chronic kidney disease (CKD). Current treatment strategies remain unsatisfactory in terms of complete renal response, prevention of relapses, CKD, and progression to end-stage kidney disease. To improve the prognosis of LN, recent data suggest that we should *(i)* modify our treat-to-target approach by including, in addition to a clinical target, a pathological target and *(ii)* switch from conventional sequential therapy to combination therapy. Here, we also review the results of recent controlled randomized trials.

## 1. Introduction

Lupus nephritis (LN) occurs in 12 to 69% of patients suffering from systemic lupus erythematosus (SLE), depending on case series [1]. Based on clinical and laboratory findings, it affects around 50% of SLE patients, while the rates of biopsy-proven LN are somewhat lower [2]. LN is more prevalent in Asian than in African or Hispanic and European patients [3].

## 2. Pathophysiology of Lupus Nephritis

Immune complexes (IC), produced in lymph nodes, spleen, or other lymphoid tissues are deposited in the glomeruli of LN patients [4]. Their detection, by direct immunofluorescence techniques on kidney biopsies, is part of the diagnosis of LN [5]. Antibodies also cross-react with glomerular antigens (DNA, histones, and nucleosomes) [6,7], in particular from the basement membrane [8]. The location of IC deposits explains the clinical phenotype. Subendothelial IC induce endothelial dysfunction and recruitment of macrophages and T cells into crescents, which also contain proliferating cells from the parietal layer of Bowman’s capsule, thereby causing the so-called “proliferative” variants. Monocytes are recruited from the blood and differentiate into CD16^+^ inflammatory macrophages. Subepithelial IC cause damage to podocytes, but pro-inflammatory cell recruitment is more limited because the glomerular basement membrane prevents contact with the intravascular space. There is less glomerular inflammation, thereby less kidney failure. By contrast, the enlargement of basement membrane pores explains the (usually massive) proteinuria. Those proliferative variants with subendothelial immune deposits correspond to class III/IV LN, based on the International Society of Nephrology/Renal Pathology Society (ISN/RPS) classification [9], while subepithelial immune deposits correspond to class V LN. Beyond this first wave of immune effectors which mainly target the glomeruli, LN is also characterized by tubulointerstitial lesions which do not result from passive deposition of IC but are part of an adaptive immune response. Myeloid and plasmacytoid dendritic cells and lymphocytes are recruited in the tubulointerstitium. Antigens can be presented to T cells. T–B cell interactions promote differentiation of B cells into plasma cells that secrete antibodies against renal antigens, such as vimentin [10]. Tubulo-interstitial inflammation and hypoxia induce metabolic dysfunction and atrophy of tubular cells [11]. The lupus-affected kidney not only is the passive victim of an innate immunological attack against the glomeruli, but also participates as an actor to promote a pan-nephritis.

## 3. Clinical and Pathological Diagnosis of Lupus Nephritis

LN, which is clinically silent for a long time, is usually revealed by proteinuria and, in proliferative variants, by abnormal urinalysis and/or renal impairment. High blood pressure, edema secondary to hypoalbuminemia, and salt and water retention may complete the clinical picture.

In case of suspicion of LN, the proteinuria threshold at which a kidney biopsy is indicated is not defined. In practice, this procedure is proposed when the proteinuria level is ≥500 mg/day. Observational data show that proteinuria between 500 and 1000 mg/day is already associated with significant kidney damage [12] and also that “low-grade” proteinuria does not exclude significant kidney injury in LN [13]. Furthermore, it is well established that early management of LN improves the prognosis of the disease [14], an additional argument in favor of an early biopsy. The most common lesion observed in LN is glomerulonephritis with immune deposits. A kidney biopsy has several goals: *(i)* to characterize the type of glomerular involvement and thereby guide immunosuppression; *(ii)* to consider other mechanisms of renal injury such as thrombotic microangiopathy or podocytopathy, which require a different therapeutic approach; and *(iii)* to assess the chronicity and therefore the irreversibility of the lesions. The discovery of tubulointerstitial nephritis is not exceptional [15] and is also associated with a worse prognosis, independent of glomerular lesions [16].

The histological description of LN is based on classification criteria defined by the ISN/RPS [9], whose main goal is to guide treatment decisions. However, the ISN/RPS classification is a simplified view of the process and does not allow, at the onset of the disease, to capture with sufficient accuracy the very patients who will progress to chronic kidney disease (CKD). Thus, it has been indeed demonstrated that the long-term prognosis of classes III, IV-S, and IV-G LN is similar [17]. The pitfall probably stems from the glomerulocentric nature of this classification, which does not take into account tubulointerstitial lesions, known to be a major driver of renal impairment [18]. A recent revision of the ISN/RPS classification recommends their inclusion [19].

## 4. Treatment of Lupus Nephritis

### 4.1. Global Therapeutic Strategy

The ultimate goal of LN treatment is to prevent nephron loss and, thereby, CKD, especially end-stage kidney disease (ESKD). Since the risk of kidney impairment is greater in patients with proliferative LN, immunosuppressants (IS) play a pivotal role in the treatment of ISN/RPS Classes III and IV LN. To prevent CKD, the therapeutic goal in the short term is to achieve complete, at least partial, resolution of the clinical and laboratory signs of LN. At diagnosis, the kidneys are already severely damaged by glomerular deposits of immune complexes and tubulointerstitial inflammation. Patients must therefore be treated with powerful and promptly efficacious anti-inflammatory agents, such as glucocorticoids (GCs), combined with another IS agent to interrupt autoimmune processes. Immunosuppression should be maintained for several years. The standard immunosuppressive strategy consists of a formerly called “induction phase” followed by a “maintenance phase”. These terms are now replaced by “initial treatment” and “subsequent treatment”, which are more appropriate.

### 4.2. Current Therapeutic Recommendations

Th therapy of LN is based on the joint recommendations of the European League Against Rheumatism (EULAR) and the European Renal Association-European Dialysis and Transplant Association (ERA-EDTA) [20]. Proposals are based on the results of controlled randomized trials summarized in Table 1. The decision to treat LN and the choice of the treatment regimen is based on the ISN/RPS classification criteria [16].

In addition to optimal nephroprotection (angiotensin-converting enzyme inhibitor (ACEI) or angiotensin receptor blocker (ARBs)) and hydroxychloroquine (HCQ; 5 mg/kg/day, except in cases of severe kidney impairment for which the dose is reduced), the initial treatment of classes III/IV (±V) LN includes GCs administered intravenously (IV) (total dose of 500 to 2500 mg of IV methylprednisolone) and then orally (prednisolone 0.3–0.5mg/ kg/d until week 4, reduced to ≤5–10 mg/d at month 3), in combination with either mycophenolate mofetil (MMF; target dose 2 g/d) or IV cyclophosphamide (CY), according to the Euro-Lupus regimen (EL; 6 fortnightly doses of 500 mg IV CY) [21]. An alternative is a combination of MMF (dose of 1 g) with a calcineurin inhibitor (CNI; tacrolimus 4 mg/day). In case of acute kidney injury, cellular crescents, and/or fibrinoid necrosis, the aforementioned regimens are also recommended, but higher doses of IV CY can also be proposed according to the National Institutes of Health (NIH) regimen [22].

Subsequent treatment, for at least 3 years, of Classes III/IV (±V) LN is based on MMF (2 g/day) or azathioprine (AZA) (2 mg/kg/day), in addition to HCQ and the lowest possible dose of oral prednisolone (2.5–5 mg/day, ideally not administered to patients with complete response) [20].

Of note, the management of GCs in LN is based more on convention than on evidence, although recent studies support the use of lower doses with the same efficacy but less secondary effects [23,24].

Beyond immunosuppression and nephroprotection, the treatment of LN also includes optimal pharmacological control of blood pressure and preventive measures to avoid the side effects of GCs, such as prescription of calcium salts, vitamin D3 supplements, immunization against pneumococcus and influenza, and exercise.

While it is true that this approach has ensured an overall survival of 80% at 5 years, the rate of complete renal response (CRR) at 6–12 months is only 20–40%, and up to 5–20% of patients will progress, often late, to ESKD, while an additional percentage will develop CKD. Even in patients achieving CRR after treatment, an increase in chronic lesions is observed on repeat renal biopsies [25]. Relapses, occurring in approximately 20–25% of patients within 3 to 5 years [26,27], constitute a significant risk factor for the development or progression of CKD [28]. Overall, the current treatment strategies remain unsatisfactory.

## 5. How to Improve the Prognosis of Lupus Nephritis?

We suggest that the outcome of LN could be improved by the adoption of a treat-to-target approach and by switching from sequential to combination therapy.

### 5.1. Treat-to-Target Approach

The treat-to-target approach is a very fashionable concept in inflammatory and auto-immune diseases. As far as LN is concerned, we can define several types of targets: clinical, pathological, or even immunological targets at the level of the tissue, i.e., the kidneys.

#### 5.1.1. Clinical Target

The best early predictor of a good long-term kidney outcome in LN is a prompt fall in proteinuria. Thus, we demonstrated that achieving a proteinuric target of <0.7 g/day after one year of treatment has a remarkable positive predictive value for a good long-term kidney outcome (94%) [32,34]. These data are robust because they were confirmed in two additional cohorts [35,36]. Alas, the negative predictive value (NPV) of achieving this target is only 31%, which means that 69% of patients not meeting the target will still (and fortunately!) achieve a good long-term kidney outcome. The challenge is therefore to identify those very patients (one-third) who do not reach the target and will suffer from kidney impairment in the long term in order to optimize their treatment, by switching to another IS or by adding a biotherapy, always after evaluating patients’ adherence to the treatment [37]. For some cases, the kinetics of the proteinuria fall provides an argument for a “wait-and-see” attitude. Thus, in nephrotic patients at diagnosis, the failure to reach the proteinuric target should be put into perspective, and the right attitude might consists in allowing a few additional months of observation before making a therapeutic decision, certainly if the kinetics of the proteinuria decrease suggests that the target is in sight.

Attempts to add other clinical parameters (kidney function, disappearance of microhaematuria) to the proteinuric target to improve the NPV have failed. Conversely, inclusion of urinalysis (red blood cells) to the target reduced sensitivity from 71 to 41%, implying that 59% of the patients who will experience a good long-term kidney outcome would not be identified at month 12 if persistent hematuria is included in the target, while only 29% would be missed if proteinuria alone is used as criteria [34,35].

Considering that urinary proteins might be more specific for nephritis than serum proteins [38], urinary biomarkers have stimulated much research, the more so as proteomic mass spectrometry has facilitated their detection [39]. Several urinary proteins have been reported as potential predictors of LN activity, in particular NGAL (neutrophil gelatinase-associated lipocalin), MCP-1 (monocyte chemoattractant protein-1), TWEAK (tumor necrosis factor-like weak inducer of apoptosis), and PTGDS (prostaglandin D synthase) [40,41,42,43,44,45,46]. Unfortunately, their presence usually correlates with proteinuria, thereby compromising their interest. Moreover, very few studies have evaluated their long-term prognostic value.

Overall, clinical parameters, readily measurable in blood or urine, will probably not help to capture patients who need treatment optimization amongst those who have not reached the proteinuric target.

#### 5.1.2. Pathological and Immunological Target

Together with other groups, we suggest that per protocol repeat kidney biopsies performed after 12 months of IS treatment might contribute to the identification of patients who require treatment intensification. Several studies have indeed shown that signs of histological activity of LN may persist in patients with CRR after IS treatment [25,47,48,49,50]. Furthermore, we recently demonstrated in a retrospective analysis performed on incident cases of LN that residual histological activity, i.e., a NIH activity index score >3, in per protocol repeat biopsies predicted subsequent renal relapse and that chronic damage, i.e., a NIH chronicity index score >3, predicted long-term kidney impairment [51]. It is worth noting that active lesions in the glomeruli mostly accounted for the association with relapses, whereas chronic damage in the tubulointerstitial compartment was found to be a more important contributor to the association with long-term renal function.

**Figure 1:**
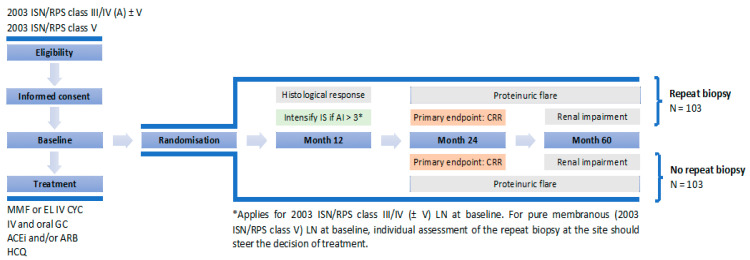
REBIOLUP flowchart.

To evaluate the value of per protocol repeat biopsy after one year of treatment, we designed a prospective international study, *REBIOLUP* (www.rebiolup.com) (accessed on 31 January 2021), illustrated in Figure 1. Patients with incident LN will be treated for one year according to current standard of care (SOC), based on shared patient’s and physician’s decision. At baseline, they will be randomized in two groups, undergoing—or not—a per protocol control kidney biopsy at one year. The control group, without re-biopsy, will be treated according to clinical parameters, whereas histologic findings will drive treatment decisions in the re-biopsy group. Thus, if the NIH activity index at re-biopsy remains superior to 3/24 [51], immunosuppressive therapy will be intensified, again according to shared patient’s and physician’s decision. The goal of the study is to demonstrate that: *(i)* the percentage of patients in CRR at 2 years (primary endpoint) will be higher in the re-biopsy group; and *(ii)* conversely, that the percentage of patients with decreased kidney function at 5 years will be lower. Should these hypotheses be confirmed, a systematic one-year repeat kidney biopsy would become an integral part of LN management, hopefully leading to a significant decrease in the number of patients suffering from CKD.

REBIOLUP will also address the question of in situ immunological response or even remission after one year of treatment, by assessing the reduction or disappearance of immune deposits, as detected by electron microscopy.

### 5.2. Combination Therapy

Based on the conclusion that many patients suffering from LN do not achieve short-term remission and experience CKD with the current regimens, we propose a new treatment paradigm, which consists in switching from sequential to combination therapy. So far, three successful combinations have been reported with CNIs (tacrolimus (TAC) and voclosporine (VOC)), anti-BlyS/BAFF belimumab, and anti-CD20 obinutuzumab.

The first reported successful combination therapy for LN was an association of MMF and TAC. Thus, a large Chinese study demonstrated that the rate of CRR at 6 months almost doubled for patients treated with a combination of MMF and TAC compared to patients given NIH IV CY, namely, a starting dose of 0.75 (adjusted to 0.5 to 1.0) g/m² of body surface area every 4 weeks for 6 months (45.9% versus 25.6%, respectively), without additional adverse effects [33]. This regimen has been reported mainly for Asian patients, and the findings may not be generalizable to other populations. After 6 months of treatment, patients randomized in the MMF/TAC arm stayed on the same regimen (although the daily TAC dose was reduced from 4 to 2 mg), while patients who received NIH IV CY were treated with AZA. Interestingly, the two groups reached a similar rate of CRR after 18 months [52], thereby suggesting that the anti-proteinuric effect of TAC may be responsible for most of the early benefit noticed at month 6.

Interesting results were observed with the combination of MMF and VOC. VOC is a more potent CNI than cyclosporine A, has more predictable pharmacodynamics (thereby avoiding repeated drug monitoring), and presents a faster elimination of metabolites (thereby is likely less responsible for adverse events). In a phase 2 (*AURA*) and a phase 3 trial (*AURORA*), where steroids were very promptly tapered, it was shown that, compared to MMF alone, the combination of MMF and VOC induced a higher CRR at 6 and 12 months (in the AURA trial, 27.3% versus 32.6% and in the AURORA trial, 22.5% versus 40.8%, in the two groups, respectively) [53,54,55]. Despite these results being clinically significant and obtained without additional serious adverse events, most likely leading to labeling of VOC for LN by the medical agencies in the next months, some caveats must be addressed. The beneficial effect of VOC might be explained by its anti-proteinuric action through stabilization of the podocyte cytoskeleton rather than a true synergistic IS effect. This raises concerns about a rebound effect when stopping the medication. Second, only short-term results (maximum 12 months) have been reported so far. Long-term toxicity data are eagerly awaited. Recently, the U.S. Food and Drug Administration (FDA) had approved voclosporin in combination with a background immunosuppressive therapy to treat patients with active LN. It is the first FDA-approved oral therapy for LN [56].

Anti-BlyS/BAFF belimumab (BEL) is the first biologic approved for SLE, based on several pivotal trials, such as BLISS 52 [57] and BLISS 76 [58]. In these trials, patients with major kidney involvement were excluded. Yet, analysis of the subset of patients with some degree of proteinuria revealed a benefit from BEL with respect to SOC in terms of proteinuria decrease [59], which led to the design of a controlled specific LN trial (BLISS-LN), whose results were recently released [60]. In this trial, the largest and the longest ever performed for LN, 448 patients with active LN were randomized to receive either BEL (one injection every month for two years) or placebo (PBO), as an add-on therapy superimposed on SOC. The SOC was left to the decision of the physician and the patient and consisted in MMF or EL IV CY followed by AZA. The primary endpoint, which was the primary efficacy renal response at week 104 (defined as a urinary protein-to-creatinine ratio ≤0.7 g/g, an estimated glomerular filtration rate (eGFR) no worse than 20% below the pre-flare value or ≥60 mL/min/1.73 m², and no use of rescue therapy), was reached by significantly more patients in the BEL group than in the PBO group (43% versus 32%, respectively). Intriguingly, the beneficial effect of BEL was only observed in conjunction with MMF and not with EL IV CY followed by AZA. The reasons for this difference are unclear, but the CY-treated patients were more severely affected at baseline (higher level of urinary protein, lower eGFR, lower complement concentrations, longer disease duration, and greater exposure to previous treatment for LN, suggesting a greater kidney damage accrual in patients who received CY as SOC), which might explain why these patients did not benefit from the addition of BEL. The time to renal events (ESKD, doubling of serum creatinine, death, and renal flares) was different, again favouring BEL against PBO. The side effects did not differ between between the two groups. The results of a 6-month open-label extension study performed in *BLISS LN* completers should be released very soon and will tell us whether further improvement is observed with time with BEL treatment. Interestingly, some real-life observations indicated a possible appearance of active LN during treatment with BEL in patients who did not have a renal phenotype of SLE prior to BEL initiation [61,62,63,64,65].

Obinutuzumab (OBI) is an anti-CD20 monoclonal antibody that has been glycoengineered to increase antibody-dependent cytotoxicity. It has a type II binding conformation which leads to a greater direct cell death effect and more limited internalization of the monoclonal antibody. These characteristics result in a much more pronounced and sustained B cell depletion compared to rituximab (RTX) [66]. Since better B cell depletion, especially in the kidneys themselves, may increase the rate of CRR, OBI was tested in a small phase II trial, called *NOBILITY* [67]. The study drug (1000 mg administered on days 1, 15, 168, and 182) was compared to PBO on a MMF background and a moderate dose of promptly tapered GCs. At week 52, 76, and 104, the percentage of patients achieving CRR was higher for patients given OBI compared to those receiving PBO, reaching 35% (versus 23%) at week 76 and 41% (versus 23%) at week 104. Almost all patients still had very low peripheral B cell counts (CD19^+^ count ≤5 cells/μL) at week 52, a finding much different from that of the *LUN*AR trial where only half of RTX-treated patients had undetectable peripheral B cells after one year of treatment [68]. The side effects were comparable in both groups, without additional toxicity due to the combination therapy. Of note, *NOBILITY* is a small trial mainly performed in Hispanic patients. Confirmation might come from a global phase III trial, called *REGENCY* (ClinicalTrials.gov NCT04221477), in which six doses of OBI/PBO will be given to LN patients, with CRR as primary outcome measured at week 76.

These recent trials, *AURA*, *AURORA*, *BLISS-LN*, *NOBILITY*, testing three different drugs and using very similar definitions of CRR, led to the same conclusion: a combination therapy is superior to SOC (GC/MMF or GC/EL IV CY/AZA). The PBO response in these three trials was consistently low, between 20% and 25% CRR after 6 months to 2 years of follow-up. The effect of VOC was prompter, which is consistent with its mode of action. We eagerly await the results (expected in 2021) of the LN anifrolumab trial, called *TULIP-LN* (ClinicalTrials.gov NCT02547922) aimed at testing the efficacy of a human monoclonal antibody against type I interferon receptor subunit 1.

Of note, we decided to focus only on trials demonstrating positive results, but many other biologics were previously tested for LN. They were not developed due to side effects or ineffectiveness. Some failures are probably explained by design flaws [69].

## 6. Conclusions

We suggest to adopt a treat-to-target approach for LN to limit nephron loss and thereby prevent CKD. Per protocol kidney biopsies performed after one year of treatment might be part of this strategy, which will be tested in *REBIOLUP* (www.rebiolup.com) (accessed on 31 January 2021). The current SOC does not meet patient’s and physician’s expectations. A combination therapy, instead of a sequential therapy, might become the new paradigm, based on recent trials. Which combination should be prescribed to which patients is currently not clear, but better knowledge of patients’ molecular profiling, including at the level of the kidney itself, might possibly be helpful at the bedside.

## Figures and Tables

**Table 1:** Pivotal trials for immunosuppressant treatment of patients with lupus nephritis.

Reference	Trial	N Patients	ISN/RPS Class of LN	Treatment	Follow Up	Primary Endpoint	Results
Austin HA, NEJM 1986 [22]	NIH	107	All	High dose GC vs. NIH IV CY	Median 7 years	Time to end-stage kidney disease (ESKD)	NIH IV CY > high dose GC
Houssiau FA, A&R 2002 [21]	ELNT	90	III, IV, Vc, Vd	EL IV CY vs. NIH IV CY as induction treatment	Median 41 months	Treatment failure regarding kidney function, proteinuria, relapses	No difference observed between EL IV CY and NIH IV CY
					Mean 115 months (Houssiau, ARD 2010 [29])	Time to major event: death, ESKD, doubling of serum creatinine	No difference observed between EL IV CY and NIH IV CY
Appel GB, JASN 2009 [30]		370	III, IV et/ou V	MMF vs. IV CY as induction treatment	24 weeks	Proteinuria decrease (UPC ratio)	No difference observed between MMF and IV CY
Dooley MA, NEJM 2011 [26]	ALMS	227	III, IV ou V	AZA vs. MMF as maintenance treatment after induction by MMF or IV CY	36 months	Time to first event: death, end-stage-kidney disease, renal relapse, need of a rescue therapy	MMF > AZA Relapses: 16.4% in MMF group vs. 32.4% in AZA group
Houssiau FA, ARD 2010 [31]	MAINTAIN	105	III, IV, Vc, Vd	AZA vs. MMF as maintenance treatment after induction by EL IV CY	Mean 48 months	Time to first renal relapse	No difference observed between MMF and AZA Relapses: 19% in MMF group vs. 25% in AZA group
		87			Median 110 months (Tamirou F, ARD 2016 [32])	Time to renal flare	No difference observed between MMF and AZA Relapses: 45% in MMF group vs. 49% in AZA group
Liu Z, AIM 2015 [33]		310	III–IV ± V, V	Tacrolimus + MMF vs. NIH IV CY	24 weeks	Complete remission	Tacrolimus + MMF (45.9%) > NIH IV CY (25.6%)

NIH: National Institutes of Health, GC: glucocorticoids, ELNT: Euro-Lupus Nephritis Trial, EL: Euro-Lupus, AZA: azathioprine; MMF: mycophenolate mofetil; IV CY: intravenous cyclophosphamide, ALMS: Aspreva Lupus Assessment Study, UPC: urine protein/creatinine ratio.
